# Ectopic Expression of a Wheat WRKY Transcription Factor Gene *TaWRKY71-1* Results in Hyponastic Leaves in *Arabidopsis thaliana*

**DOI:** 10.1371/journal.pone.0063033

**Published:** 2013-05-09

**Authors:** Zhen Qin, Hongjun Lv, Xinlei Zhu, Chen Meng, Taiyong Quan, Mengcheng Wang, Guangmin Xia

**Affiliations:** The Key Laboratory of Plant Cell Engineering and Germplasm Innovation, Ministry of Education, School of Life Science, Shandong University, Jinan, Shandong, China; University of Nottingham, United Kingdom

## Abstract

Leaf type is an important trait that closely associates with crop yield. WRKY transcription factors exert diverse regulatory effects in plants, but their roles in the determination of leaf type have not been reported so far. In this work, we isolated a WRKY transcription factor gene *TaWRKY71-1* from a wheat introgression line SR3, which has larger leaves, superior growth capacity and higher yield than its parent common wheat JN177. *TaWRKY71-1* specifically expressed in leaves, and produced more mRNA in SR3 than in JN177. TaWRKY71-1 localized in the nucleus and had no transcriptional activation activity. *TaWRKY71-1* overexpression in *Arabidopsis* resulted in hyponastic rosette leaves, and the hyponastic strength was closely correlative with the transcription level of the transgene. The spongy mesophyll cells at abaxial side of leaves were drastically compacted by *TaWRKY71-1* overexpression. In *TaWRKY71-1* overexpression *Arabidopsis*, the expression of *IAMT1* that encodes a methyltransferase converting free indole-3-acetic acid (IAA) to methyl-IAA ester (MeIAA) to alter auxin homeostatic level was induced, and the induction level was dependent on the abundance of *TaWRKY71-1* transcripts. Besides, several *TCP* genes that had found to be restricted by *IAMT1* had lower expression levels as well. Our results suggest that *TaWRKY71-1* causes hyponastic leaves through altering auxin homeostatic level by promoting the conversion of IAA to MeIAA.

## Introduction

Plant type closely associates with the crop yield, and is therefore a crucial trait in crop breeding. Appropriate leaf shape is an important aspect of ideal plant type, because the three-dimensional structure of the plant leaf affects light capture, carbon fixation, and gas exchange for photosynthesis [1]. In rice, the rolled leaf is regarded as a critical element for the ideal phenotype [2], [3]. Moderate leaf rolling improves photosynthetic efficiency, accelerates dry-matter accumulation, increases yield, reduces the solar radiation on leaves, and decreases leaf transpiration under drought stress [4]. Therefore, to unravel the mechanism controlling leaf curling is beneficial for breeding crops with the desired architecture and stress tolerance.

Leaf curling is orchestrated by the complicated machineries with hormones and non-hormone factors involved. Auxin has been widely reported to play an essential role in the determination of the leaf morphology [5], [6]. Auxin signaling mutant *auxin-resistant 1* (*axr1*) results in epinastic leaves [7], while *phototrophic hypocotyl 4/auxin response factor 7* (*nph4/arf7*) produces hyponastic (up-curled) leaf phenotypes [8]. Auxin synthesis defect mutants *sporocyteless*-*Dominant I* (*spl-D*) and *petal loss-Dominant* (*ptl-D*) display a hyponastic leaves phenotype [9], but its overproduction mutants such as *rooty* [10], *sur2* [11], [12], *yucca* [13], and *iaaM* overexpression lines [14] all have epinastic leaves phenotype. Apart from the synthesis and degradation, auxin homeostasis between free auxin and its conjugates that are modified by monosaccharide, disaccharide or methyl has proved to regulate leaf curling. Overexpression of the *Arabidopsis UGT84B1*, the homologue of maize *IAGlu* that catalyzes the formation of IAA glucose ester, results in rounded, wrinkled, and curly leaves [15]. The dominant mutant *iamt1-D* of *IAMT1* (At5g55250), whose product appears to encode an IAA methyltransferase that specifically converts free IAA to methyl-IAA ester (MeIAA) *in vitro* [16], [17], causes severe hyponastic leaves [18]. In *iamt1-D*, the expression of some known leaf-curling associated genes such as several *TCP*s and *HASTY* is reduced. However, how *IAMT1*-mediated homeostasis between IAA and MeIAA is modulated is still not known.

WRKY transcription factors, named by their WRKY domain, are one of the large families of transcriptional regulators in plants. The WRKY domain is about 60 residues in length, containing the WRKYGQK residues and a zinc-finger structure at the C-terminus. The zinc-finger structure is either Cx_4–5_Cx_22–23_HxH or Cx_7_Cx_23_HxC. The WRKY transcription factors are divided into three groups based on the number of WRKY domains and their zinc fingers structure. Group I proteins contain two WRKY domains and C_2_H_2_ structures, group II proteins contain one WRKY domain and C_2_H_2_ structures, and group III proteins contain one WRKY domain and C_2_HC structures. Group II proteins were further divided into IIa, IIb, IIc, IId and IIe based on the primary amino acid sequence. WRKY proteins act as repressors or activators to play important roles in diverse processes such as plant development and response to environmental stimuli, and their functions in leaves include senescence and resistance to *Xanthomonas oryzae* (bacterial rice leaf blight) (see review [19]). The issue that whether or not WRKY transcription factors regulate plant type has not been reported so far.

To identify WRKY transcription factor that may function in leaf type (curling), we screened the microarry-based transcriptomic data between the wheat (*Triticum aestivum*) introgression cultivar SR3 and its parent common wheat JN177. SR3 has larger leaves, more vigorous growth capacity, higher yield as well as superior salt tolerance than JN177 [20]. We isolated a wheat WRKY transcription factor, *TaWRKY71-1*, specifically expressed in leaves with higher transcription level in SR3 than in JN177. Its overexpression in *Arabidopsis* resulted in hyponastic leaf phenotype, promoted the expression of *IAMT1* and several *TCP* genes, and tightened spongy tissue cells. These findings indicate that *TaWRKY71-1* resulted in hyponastic leaves by shifting auxin homeostasis from IAA to MeIAA.

## Materials and Methods

### Plant Materials

The wheat introgression line SR3 that was selected from asymmetric somatic hybrids between a bread wheat cultivar JN177 and *Thinopyrum ponticum* was used in this study [20]. Seeds of SR3 were germinated in distilled water at 25°C and 60% humidity under a photoperiod of 16 h/8 h for seven days, and then uniform seedlings were transferred into half-strength Hoagland’s liquid medium under the same condition for another seven days. Leaves and roots of fourteen-day-old seedlings with similar height were collected separately and stored in liquid nitrogen for RNA extraction and further analysis.

### Isolation and Sequence Analysis of *TaWRKY71-1* from Wheat

The sequence of the selected probe in the microarry was subjected to BLASTn against wheat EST database at NCBI, and all matched EST sequences were assembled into a contig sequence using the CAP3 package. According to the contig sequence (designated as *TaWRKY71-1* for it was an allelic gene of *TaWRKY71* isolated from wheat cultivar Chinese Spring), the gene-specific primer pairs covering the open reading frame (ORF) were designed (forward primer: 5′-CAGCGTCCATTCCATTACAGTAG-3′; reverse primer: 5′-CGTCTTCGTCGTTGGTCAGG-3′). Then the ORF and the genomic sequence of *TaWRKY71-1* were obtained from cDNA and genomic DNA of SR3, respectively, by PCR.

### Isolation of *TaWRKY71-1* Promoter and Prediction of *cis*-elements

The promoter sequence of *TaWRKY71-1* was predicted by BLAST the Chinese Spring draft genome assembly (http://www.cerealsdb.uk.net/CerealsDB/Documents/DOC_search_reads.php) with the genomic sequence of *TaWRKY71-1* as query. According to the predicted promoter sequence, the specific primers (the forward primer 5′-CGTTTTTGAAAGAGTGAGTCGAGC-3′ and the reverse primer 5′-CGGCTGGTTGTTGTAGTGGTTATTGC-3′) were designed, and *TaWRKY71-1* promoter sequence was amplified from genomic DNA. *cis*-elements in the promoter sequence were analyzed using Plant-CARE available online (http://bioinformatics.psb.ugent. be/webtools/plantcare/html/).

### Subcellular Localization of TaWRKY71-1

*TaWRKY71-1* ORF without termination codon was amplified by PCR with primers containing *Bgl*II and *Hind*III sites (underlined) at their 5′ ends, 5′-AGATCTCAGCGTCCATTCCATTACAGTAG-3′ and 5′-AAGCTTATTTATGTCCCTGGTCGGCGAT-3′. After being verified by sequencing, the fragment was introduced into pBI221 vector, creating an in-frame fusion *TaWRKY71-1::GFP* gene under the control of CaMV 35S promoter. The fusion construct (pBI221-*TaWRKY71-1::GFP*) and control vector (pBI221-*GFP*) were transformed into *Arabidopsis* protoplasts [21] and onion epidermis cells [22], respectively. After incubation at 21–23°C for 16 h, GFP was detected by a confocal fluorescence microscopy (Zeiss LSM700).

### Transcriptional Activation Analysis

For transcriptional activation analysis, the full-length of *TaWRKY71-1* cDNA was amplified using the following primers, which possess *EcoR*I and *Bgl*II sites (underlined), 5′-GAATTCATGGATCCATGGGTCAGCA-3′ and 5′-AGATCTCGTCTTCGTCGTTGGTCAGG-3′ to build the pGBKT7-*TaWRKY71-1* vector (Clontech) containing the GAL4 DNA binding domain. The pGBKT7*-TaWRKY71-1* and pGBKT7 (negative control) were transformed into yeast strain AH109. The transformed yeast cells were selected on synthetic defined (SD) plates without Trp, and analyzed on SD plates without Trp, His and Ade.

### Generation of *TaWRKY71-1* Overexpression *Arabidopsis* Lines

The ORF of *TaWRKY71-1* was cloned into the *Xba*I and *Sma*I sites of pSTART vector to create the pSTART*-TaWRKY71-1* construct driven by CaMV35S promoter using the forward primer 5′-TCTAGACAGCGTCCATTCCATTACAGTAG-3′ and the reverse primer 5′-CCCGGGCGTCTTCGTCGTTGGTCAGG-3′. The pSTART*-TaWRKY71-1* construct in *A. tumefaciens EHA105* was transformed into *Arabidopsis* (Columbia-0) using floral dipping method [23], and the transformed *Arabidopsis* was grown in soil under a 16 h/8 h photoperoid, 60∼80% relative humidity, 21–23°C regime. Seeds of transformed *Arabidopsis* were screened in 1/2 MS agar plate containing kanamycin and cephalosporin in dark at 4°C for 72 h followed by under a 16 h/8 h photoperoid, 60∼80% relative humidity, 21∼23°C regime for two weeks. Homozygous T3 progeny bred from a T2 population which segregated in the correct ratio 3∶1 were selected and confirmed by semi-quantitative RT-PCR (sqRT-PCR) with total cDNAs as template, which was used for further analysis.

### RNA Extraction and Gene Expression Assay

Total RNA of wheat and *Arabidopsis* seedling samples was extracted using TRIZol reagent (Takara), and the first strand of cDNA was prepared using RNAiso plus kit (Invitrogen) according to the manual. sqRT-PCR for gene expression assay was performed in a 20µL reaction volume with the thermal cycling procedure that denaturation at 95°C for 5 min, followed by 28/38 cycles of 94°C/30 s, 55°C/30 s, 72°C/45 s, with a final extension of 72°C/5 min. Real time quantitative PCR (qRT-PCR) for *Arabidopsis* marker gene expression assay was performed in a 10µL reaction volume with the thermal cycling procedure that denaturation at 95°C for 2 min, followed by 40 cycles of 94°C/10 s, 60°C/10 s, 72°C/20 s, and then melting curve. A constitutively expressed wheat *TaACTIN* (AB181991) and *Arabidopsis TUBLIN* (AT5G62690) were used as internal reference for sqRT-PCR, and *Arabidopsis ACTIN2* (AT3G18780) for qRT-PCR. sqRT-PCR primers were: *TaWRKY71-1*, forward 5′-CAGTTCACCGACATGGTCAC-3′, reverse 5′-GTCGCCACGAGTATGGTCTT-3′; wheat *TaACTIN*, forward 5′-GTTCCAATCTATGAGGGATACACGC-3′, reverse 5′-GAACCTCCACTGAGAACAACATTACC-3′; *Arabidopsis TUBLIN*, forward 5′-CTCAAGAGGTTCTCAGCAGTA-3′, reverse 5′-TCACCTTCTTCATCCGCAGTT-3′. Primers used for qRT-PCR analysis of *Arabidopsis* genes were: *TCP3*, forward 5′-TAGCTTCAACGCAACAGAGC-3′, reverse 5′-GGTTCTGTGTATTGCCTCGTG-3′; *TCP4*, forward 5′-CACGACGGTCTCACTCACAA-3′, reverse 5′-AATCTAAGTCAAGCTTCAATGTGC-3′; *TCP10*, forward 5′-CCACGGAGAAGAAGCTACTCA-3′, reverse 5′-TCATCATGAATTTGAACCTCCA-3′; *TCP17*, forward 5′-TGGCTCTTAGAAGTTGCCAAA-3′, reverse 5′-TGAAAACCAGGTGGGAATTG-3′; *TCP24*, forward 5′-GCTCATGACAAGAATCTGAAGAAA-3′, reverse 5′-TGTTGCAGTGATAAACTTTTGAATTT-3′; *ARF8*, forward 5′-GACATGAAGCTGTCAACATCTGG-3′, reverse 5′-TAGGTTGCTTACTCGGTATCC-3′; *IAMT1*, forward 5′-CAACAACGGCATACAAACGG-3′, reverse 5′-TCTCTGCTGCTACCAAACCC-3′; HASTY, forward 5′-AGAGGGCAGAGCAAAGGTTC-3′, reverse 5′-TTCAAGAGGAAACCCACCATAG-3′; *ACTIN2*, forward 5′-CCGGTATTGTGCTGGATTCT-3′, reverse 5′-TTACAATTTCCCGCTCTGCT-3′. Three biological replicates and three technical replicates for each sample were performed.

### Leaf Paraffin Section Analysis

For paraffin section analysis, the mature rosette sixth leaves were collected and fixed in Carnoy’s solution (60∶30:10, ethanol:chloroform:glacial acetic acid, v/v/v) for 12 h. Samples were washed with 60% (v/v) ethanol, immersed in 30% (v/v) hydrofluoric acid for approximately 7 to 10 d, and then dehydrated with a graded ethanol series and embedded in paraffin. Sections (approximately 8–10 mm thick) were cut with a microtome (Leica), stained with 1% (w/v) safranin O (Amresco) and 1% (w/v) fast green FCF (Merck), examined with a microscope and photographed.

## Results

### TaWRKY71-1 Encoded a Group IIa WRKY Transcription Factor

**Figure 1:**
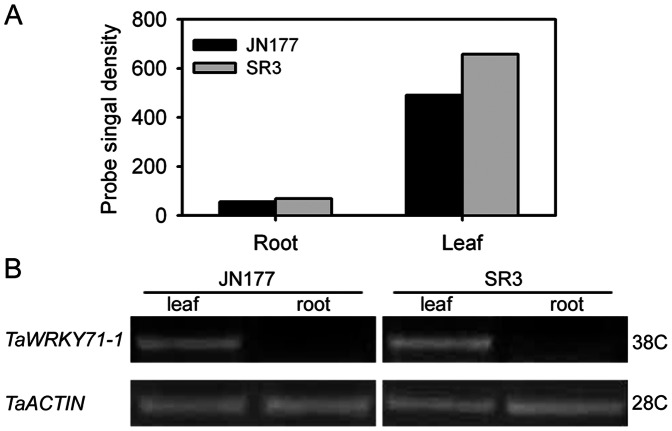
*TaWRKY71-1* are specifically transcribed in wheat leaves with a higher expression level in SR3. A: The hybridization signals of the probe referring to *TaWRKY71-1* in the microarray using wheat seedlings harvested at three-leaf stage. Data present as mean ± standard deviation. B: The sqRT-PCR using two-week old wheat seedlings. A wheat actin gene was used as the internal reference. JN177, a common wheat cultivar; SR3, a wheat introgression cultivar selected from somatic hybrids between JN177 and its close relative grass *Thinopyrum ponticum* via asymmetric somatic hybridization. 28C, 28 cycles; 38C, 38 cycles.

**Figure 2:**
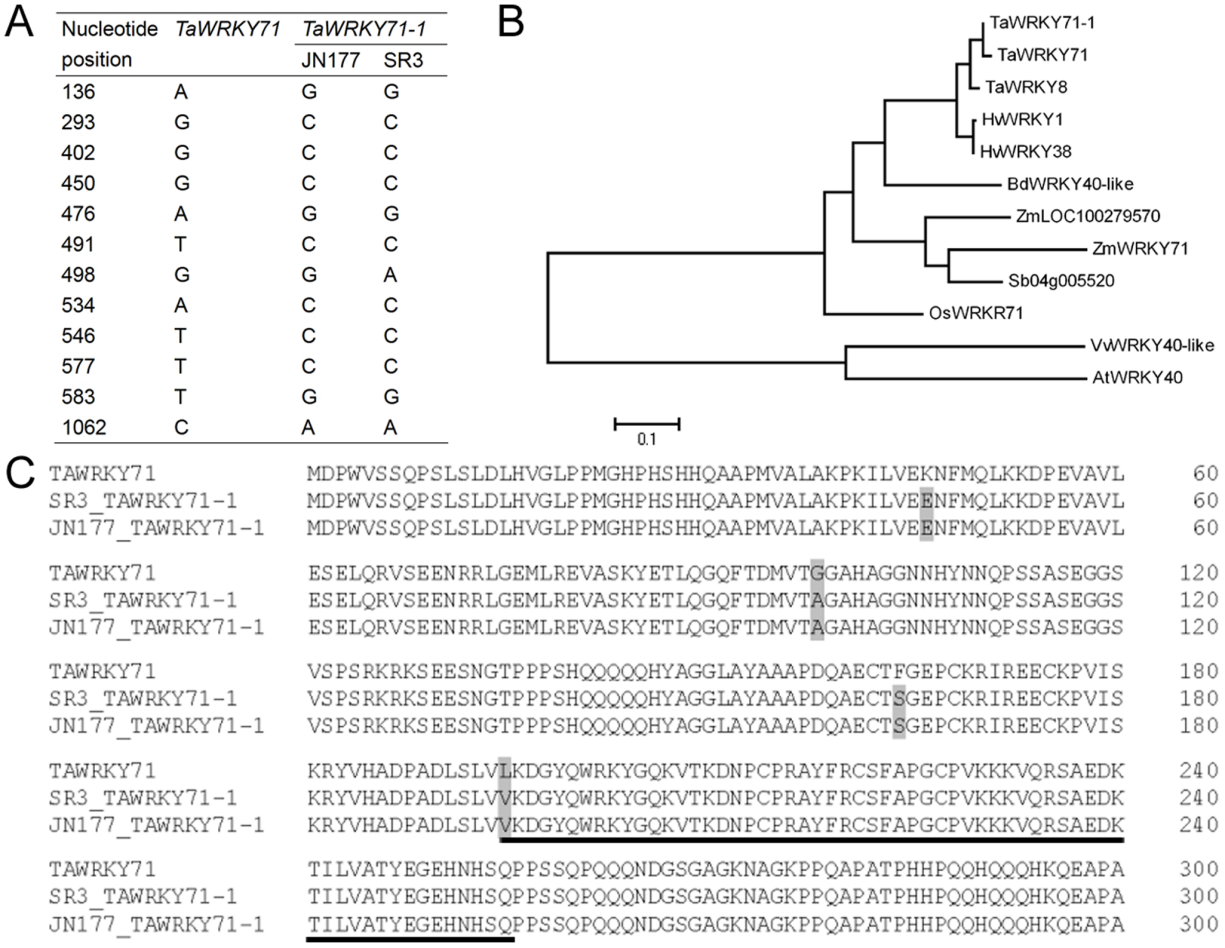
*TaWRKY71-1* encodes a group IIa of WRKY transcription factor. A: the statistics of single nucleotide polymorphisms of *TaWRKY71-1* from SR3 and JN177 and its homologous gene *TaWRKY71* from wheat cultivar Chinese Spring. B: The phylogenetic analysis of TaWRKY71-1 and its aligned WRKY transcription factors from NCBI at the amino acid level. C: Sequence alignment of TaWRKY71-1 from SR3 and JN177and its homologous gene TaWRKY71 from Chinese Spring. Underlined peptide sequence is a WRKY domain. *Ta, Triticum aestivum; Hv, Hordeum vulgare; Bd, Brachypodium distachyon; Zm, Zea mays; Sb, Sorghum bicolor; Os, Oryza sativa; Vv, Vitis vinifera; At, Arabidopsis thaliana.*

To identify wheat WRKY transcription factors that may account for leaf type, we screened the probes referring to WRKY transcription factor from the microarray data of SR3 and JN177 seedlings at three-leaf stage, and found a probe that predominantly transcribed in leaves and had a higher expression level in SR3 than in JN177 (Fig. 1A). The gene referring to the probe was isolated from SR3, and it consisted of a CDS with 1068 nucleotides in length that was separated by a 107-nucleotide intron. This gene had high identity to *TaWRKY71* (Genbank accession ID: 100049023) of wheat cultivar Chinese Spring with only twelve single nucleotide polymorphisms (SNPs) (Fig. 2A), indicating that it was an allelic gene of *TaWRKY71*, so this gene was termed as *TaWRYK71-1*. *TaWRKY71-1* encoded a protein of 355 amino acid residues with a predicted molecular weight of 38.79 kDa and a theoretical pI of 7.72. Among the characterized proteins, TaWRKY71-1 showed the most homology with TaWRKY71 (Fig. 2B). TaWRKY71-1 possessed a single WRKY domain (Fig. 2C), and based on the primary amino acid sequence of this domain, it has been assigned to group IIa of WRKY transcription factors.

Twelve allelic variations (SNPs) in the CDS region between *TaWRYK71-1* and *TaWRYK71* (Fig. 2A) brings about four substitutions of amino acid residues between their deduced peptide sequences (one substitution occurred in WRKY domain) (Fig. 2C). SR3 genome had taken place whole-genome-scale genetic (DNA sequence) and epigenetic (DNA methylation) variations during asymmetric somatic hybridization ([24], unpublished data). To determine whether these SNPs came from the natural mutation between Chinese Spring and JN177 or from the variation during asymmetric somatic hybridization, we isolated the homologous sequence of *TaWRKY71-1* from JN177. Among the twelve SNPs, eleven SNPs came from the natural mutation (present between *TaWRKY71* and JN177 *TaWRKY71-1*, but not between SR3 and JN177), and the other one came from asymmetric somatic hybridization (present between SR3 and JN177, but not between *TaWRKY71* and JN177 *TaWRKY71-1*) (Fig. 2A). The SNP induced by asymmetric somatic hybridization was synonymous nucleotide substitution, which did not result in the change of amino acid residue (Fig. 2C).

### TaWRKY71-1 Expression and its Promoter Analysis

To confirm the microarray result, *TaWRKY71-1* expression profile was assayed with sqRT-PCR. *TaWRKY71-1* transcribed in the leaves of two-week-old seedlings in both SR3 and JN177, but its transcript was not detected in roots (Fig. 1B). Moreover, there had higher expression level in SR3 than in JN177 (Fig. 1B). Our unpublished data indicated that some genes with differential transcriptional levels between SR3 and JN177 partially owe to the genetic and epigenetic variations in their promoter regions. To know whether or not these variations also account for the differential expression of *TaWRKY71-1* between two cultivars, the 739bp fragment present upstream of *TaWRKY71-1* coding sequence was isolated from SR3 and JN177 genomic DNA. The promoter sequences between two cultivars were absolutely identical, and their methylation frequencies were also comparable (data not shown). This suggests that the differential expression of *TaWRKY71-1* between SR3 and JN177 is modulated by its upstream regulatory factors rather than the promoter sequence itself.

There had a number of putative *cis*-acting elements involved in light response (Box I, G-box, GATA-motif, Sp1, TCCC-motif), gibberellin response (GARE-motif), meristem specific activation (CCGTCC-box), and lignin synthesis (AC-I) (Table 1), which closely relate to plant development and growth. Along with its higher expression in SR3, it could be implied that *TaWRKY71-1* is correlated with vigorous growth and large leaf of SR3. Besides, the promoter also possessed the elements that are associated with the response to hormones such as ABA (ABRE) and jasmonic acid (CGTCA-motif, TGACG-motif) as well as to fungal elicitor and pathogen (Box-W1, W box). These hormones have proved to participate in plant development and response to environmental stimuli, suggesting *TaWRKY71-1* might be involved in these processes. Consistent with this suggestion, *TaWRKY71-1* was found to be induced by several abiotic stresses (data not shown).

**Table 1:** The predicted *cis*-elements in *TaWRKY71-1* promoter sequence.

Element	Position (Strand)^a^	Core sequence	Function
A-box	171 (+), 344 (+)	CCGTCC	*cis*-acting regulatory element
ABRE	411 (+)	CCTACGTGGC	*cis*-acting element involved in the abs*cis*ic acid responsiveness
AC-I	176 (+), 177 (+)	CCCACCTACC	lignin synthesis associated
Box I	4 (−)	TTTCAAA	light responsive element
Box-W1	106 (+)	TTGACC	fungal elicitor responsive element
CCAAT-box	186 (−)	CAACGG	MYBHv1 binding site
CCGTCC-box	171 (+), 344 (+)	CCGTCC	*cis*-acting regulatory element related to meristem specific activation
CGTCA-motif	271 (+)	CGTCA	*cis*-acting regulatory element involved in the MeJA-responsiveness
G-box	364 (+), 561 (+)	CACGAC	*cis*-acting regulatory element involved in light responsiveness
GARE-motif	243 (−)	TCTGTTG	gibberellin-responsive element
GATA-motif	98 (−)	AAGGATAAGG	part of a light responsive element
Sp1	178 (+), 339 (+), 182 (+), 481 (+)	CC(G/A)CCC	light responsive element
TCCC-motif	629 (+)	TCTCCCT	part of a light responsive element
TGACG-motif	271 (−)	TGACG	*cis*-acting regulatory element involved in the MeJA-responsiveness
W box	106 (+)	TTGACC	pathogen- or elicitor-responsive

a Position, the nucleotide number upstream the start codon ATG; Strand,+and - present the element exists in sense strand and antisense strand, respectively.

### TaWRKY71-1 Localized in the Nucleus

**Figure 3:**
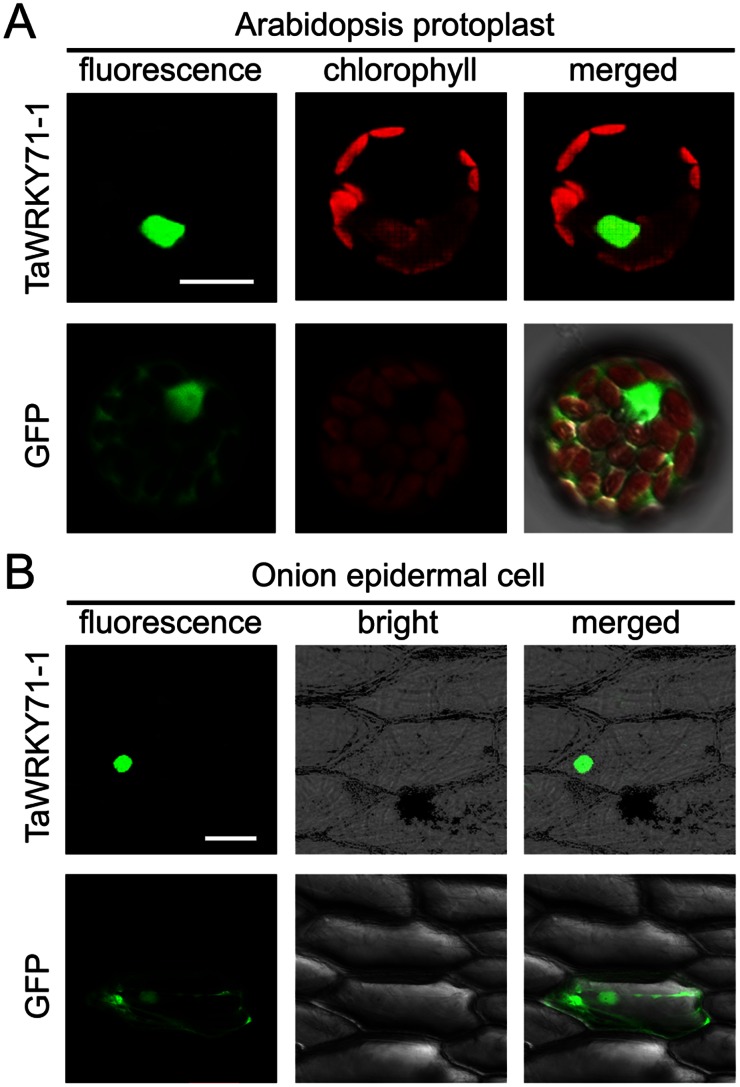
TaWRKY71-1 localizes in the nucleus. Transiently expressed TaWRKY71-1 in-frame fused with GFP in *Arabidopsis* mesophyll protoplasts (A) and onion epidermis cells (B). Bar in A 10µm and in B 100µm.

TaWRKY71-1 protein was predicted to localize in the nucleus using WoLF PSORT (http://wolfpsort.org/). To confirm this at the cellular level, the transient expression vector pBI221 expressing in-frame TaWRKY71-1:GFP fusion protein (pBI221-*TaWRKY71-1-GFP*) and the negative control (pBI221-*GFP*) were transformed into *Arabidopsis* protoplasts and onion epidermal cells. In both systems, TaWRKY71-1-GFP fusion proteins were restricted to the cell nucleus while the control GFP protein was observed throughout the entire cell (Fig. 3). This result shows that TaWRKY71-1 is a nuclear protein, further indicating that TaWRKY71-1 is a transcription factor.

### TaWRKY71-1 had no Transcriptional Activation Activity in Yeast

**Figure 4:**
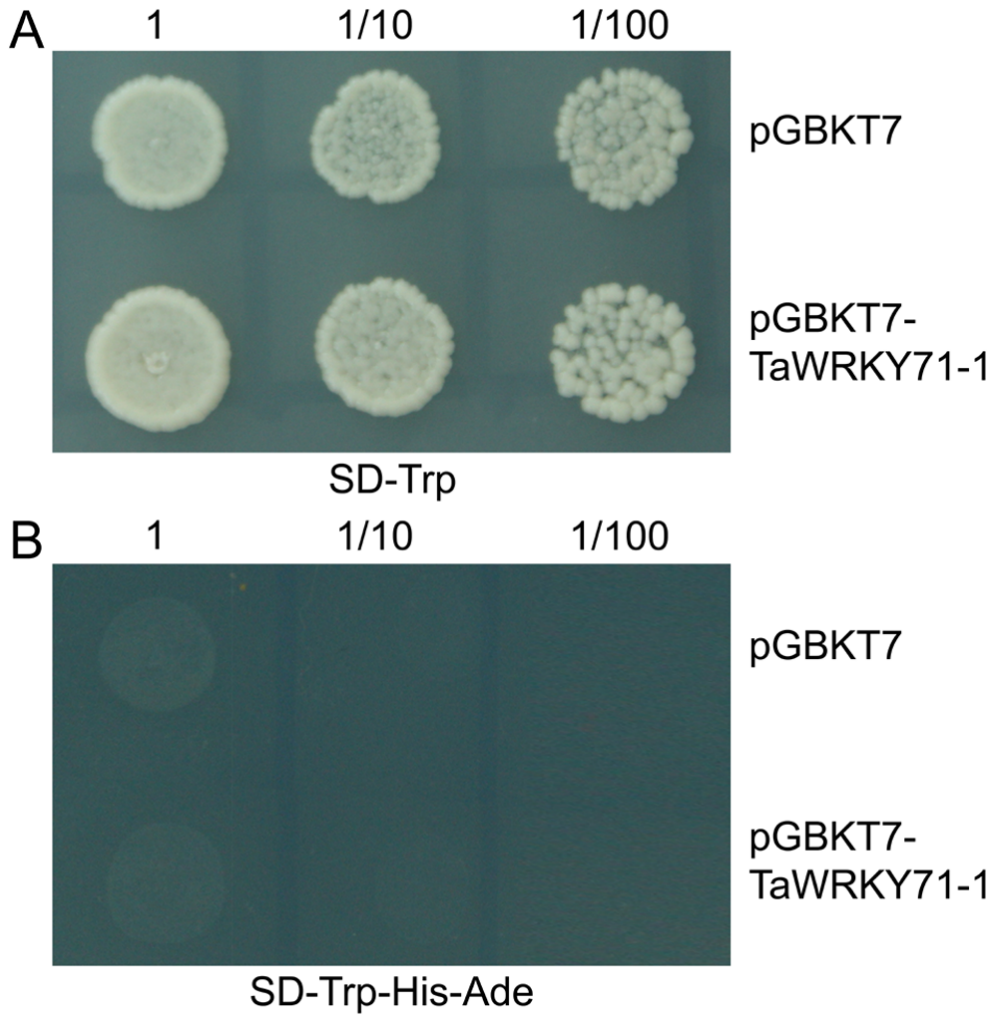
TaWRKY71-1 has no transactivation activity. The full length ORF of *Ta*WRKY71-1 was constructed into pGBKT7, and transformed yeasts were selected on both SD-Trp and SD-Trp-His-Ade media, respectively.

To determine the transcriptional activation activity of TaWRKY71-1 protein, a fusion protein containing a GAL4 DNA binding domain (GAL4-BD) and the whole ORF of *TaWRKY71-1* was constructed (pGBKT7*-TaWRKY71-1*). The fusion plasmid pGBKT7*-TaWRKY71-1* and pGBKT7 (negative control) were respectively transformed into yeast strain *AH109* containing the upstream activating sequence (UAS), which could be specifically bound by GAL4 binding domain. All transformants containing pGBKT7*-TaWRKY71-1* or pGBKT7 grew well on selective medium without tryptophan (SD-Trp), whereas they did not grow on selective medium without tryptophan, histdine and adenine (SD-Trp-His-Ade) (Fig. 4). These results indicated that TaWRKY71-1 indeed could not transactivate the expression of both reporter genes *HIS3* and *ADE2* as the negative control in yeast, suggested that TaWRKY71-1 may not be a transcriptional activator.

### *TaWRKY71-1* Overexpression in *Arabidopsis* Raised Hyponastic Leaves

**Figure 5:**
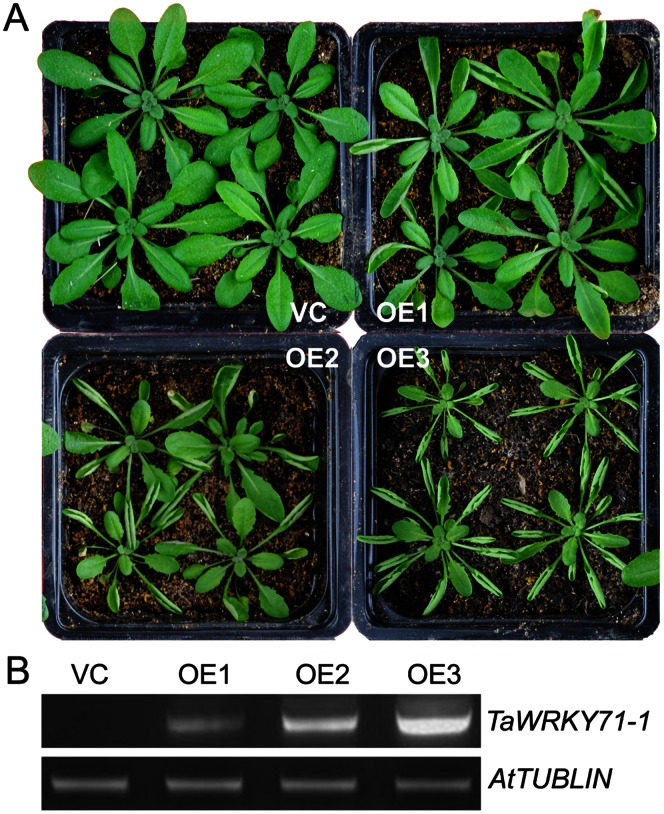
*TaWRKY71-1* overexpression results in hyponastic leaves in *Arabidopsis*. A: Phenotypic comparison of four-week old *Arabidopsis* seedlings. B: sqRT-PCR of transgenic *TaWRKY71-1* in *Arabidopsis*, and an *Arabidopsis* tublin gene was used as the internal reference. Col-0, the ecotype Columbia-0; VC, Vector control; OE1–3, three independent transgenic *Arabidopsis* lines overexpressing *TaWRKY71-1*.

*TaWRKY71-1* was transformed into *Arabidopsis* for checking its role in leaf development, and more than ten independent transgenic overexpression (OE) lines were obtained. These OE lines and empty vector control line (VC) were comparable with respect to the growth (plant size and height) and reproduction (flowing time, seed size and yield) phenotypes (data not shown). The rosette leaves of OE plants appeared indistinguishable from those of the VC plants when they first emerged, but their mature leaves obviously curled upward with different extents (Fig. 5A, data not shown). According to the curvature, the hyponastic leaves of these transgenic lines were classified into slight, moderate and drastic grades, which were illustrated by three OE lines OE1, OE2 and OE3, respectively (Fig. 5A). sqRT-PCR showed that the transcriptional level of *TaWRKY71-1* was the lowest in the lines (OE1) with the slight grade of hyponastic leaves, but the highest in the lines (OE3) with the drastic grade of hyponastic leaves (Fig. 5B; data not shown), which well coincided with the strength of leaf hyponasty. These findings demonstrate that *TaWRKY71-1* plays an important and direct role in modulating leaf hyponasty.

**Figure 6:**
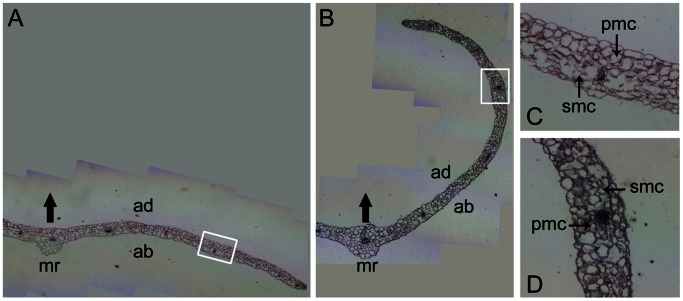
*TaWRKY71-1* overexpression tightens spongy mesophyll tissue by the paraffin section assay. A, B: Transverse sections of middle part of the 6^th^ mature rosette leaves of VC (A) and OE3 (B) lines. C, D: The enlarged view of white pane-labeled section in panels A and B, respectively. VC, Vector control; OE3, an independent transgenic *Arabidopsis* lines overexpressing *TaWRKY71-1*. Arrows in A and B show the adaxial direction. mr, main rib; ad, adaxial side; ab, abaxial side; pmc, palisade mesophyll cells; smc, spongy mesophyll cells.

To elucidate how *TaWRKY71-1* functions leaf hyponasty, the transverse sections of middle part of the mature rosette leaves were observed (Fig. 6). The cell sizes and densities of upper epidermis between the OE and VC lines both had no significant difference, as was found in lower epidermis as well. The palisade mesophyll cells at adaxial side had comparable sizes and densities between OE and VC leaves. In comparison with those of the VC leaves, the spongy mesophyll tissue cells at abaxial side of the OE leaves had similar size, but they tightened compactly with larger distribution density.

### *TaWRKY71-1* Altered the Expression of some Leaf Hyponasty Associated Genes

**Figure 7:**
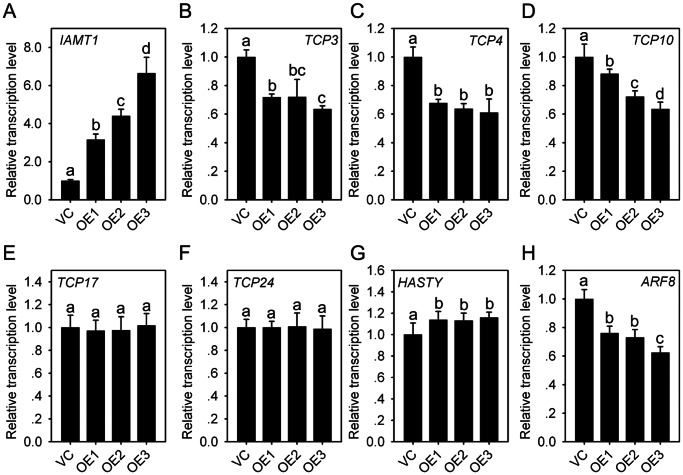
***TaWRKY71-1*** **alters the expression of** ***IAMT1*** **-mediated leaf curvature genes.** VC, Vector control; OE1–3, Three independent transgenic *Arabidopsis* lines overexpressing *TaWRKY71-1*. The leaves of four-week old *Arabidopsis* seedlings were harvested for real-time PCR. *Arabidopsis* actin gene *ACTIN2* was used as the internal reference. The graphs represent one of the same results from three independent experiments. Data present as mean ± standard deviation. Columns without the same lowercase letter are significantly different using the lowest significant difference test of one-way ANOVA (*P*<0.05).

To primarily elucidate the molecular mechanism of *TaWRKY71-1* in leaf hyponasty, the expression profiles of some genes which are involved in regulating leaf hyponasty development were examined. *IAMT1* was upregulated in the OE lines, and its induction level is the lowest in OE1 and the highest in OE3 (Fig. 7A). Among four *TCP* genes that proved to participate leaf hyponasty [25] and to be restricted by *IAMT1* [18], *TCP3, TCP4* and *TCP10* were downregulated in the OE lines (Fig. 7B–D), whereas *TCP24* was not influenced by *TaWRKY71-1* (Fig. 7F); *TCP17* that is not disturbed by *IAMT1* showed constant expression level between VC and OE lines as well (Fig. 7E). Opposite to *IAMT1* dominant mutant, *TaWRKY71-1* overexpression enhanced but did not decrease the expression of *HASTY*. Other leaf hyponasty development associated genes, *AGAMOUS*, *AXR*, *BDL*, *CLF*, *DLF*, *KANADI*, *PEAPOD*, *PTL*, *SHY*, *AS1*, *AS2*, *KAN2*, all had similar expression level between the VC and OE lines by sqRT-PCR (data not shown). Besides, *ARF8*, an auxin response factor that is induced by endogenous auxin [26], was also downregulated (Fig. 7G). Moreover, alike *IAMT1*, the change in expression strength of *TCP10*, *ARF8* as well as *TCP3* and *TCP4* among three OE lines was closely correlated with the transcription levels of *TaWRKY71-1*, reasonably revealing the direct regulation of IAA-MeIAA homeostasis by *TaWRKY71-1* during leaf hyponasty development.

## Discussion

Here, we isolated a wheat *TaWRKY71* allelic gene *TaWRKY71-1* with four amino acid residue substitutions from a wheat introgression cultivar SR3. TaWRKY71-1 contains a typical WRKY domain and localizes in the nucleus (Fig. 2C; Fig. 3), indicating that it is a WRKY transcription factor. However, TaWRKY71-1 has no transcriptional activation activity in yeast (Fig. 4). Along with the finding that OsWRKY71, a homologous protein of TaWRKY71-1, did not show transactivation activity in yeast and rice cells [27], [28], it could be suggested that TaWRKY71-1 may serve as a transcriptional suppressor.

Ectopic overexpression of *TaWRKY71-1* in *Arabidopsis* results in hyponastic leaves (Fig. 5). MeIAA is a crucial component in the auxin pool, which appears to determine the homeostatic level of auxin that is suggested to play important roles in the regulation of leaf hyponasty [15]. *IAMT1* functions in the alteration of auxin homeostasis from IAA to MeIAA [16], [17], and its *iamt1-D* dominant mutant (overexpression) results in a hyponastic leaf phenotype [18]. Importantly, in comparison with the wild-type, the heterozygous *iamt1-D* displays partially hyponastic leaves, but the homozygous *iamt1-D* has dramatically hyponastic leaves, showing the effect of *IAMT1* on leaf hyponasty depends on its mRNA abundance. Consist with this, transgenic *Arabidopsis* plants with higher *TaWRKY71-1* transcription level possess more obviously hyponastic leaves (Fig. 5), and their *IAMT1* induction extent is more significant as well (Fig. 7). Moreover, increased expression of *IAMT1* leads to down-regulation of leaf developmental genes *TCP3*, *TCP4*, *TCP10*, and *TCP24*; the higher the *IAMT1* expression level, the lower the *TCP* mRNA steady state level [18]. These *TCP* genes are highly similar to the snapdragon curvature regulation gene *CINCINNATA*, and have been shown to participate in regulating leaf curvature development [25], so it appears that *IAMT1* causes the hyponastic leaf phenotypes through decreasing the transcription of *TCP*s. Following the finding in *iamt1-D*, the expression levels of *TCP3*, *TCP4*, and *TCP10* are down-regulated in *TaWRKY71-1* overexpression *Arabidopsis*, and the downregulation strength is dependent on the mRNA abundance of *TaWRKY71-1* (Fig. 7). These data provide a strong evidence for that the role of *TaWRKY71-1* in leaf hyponasty is achieved via altering auxin homeostatic level by promoting IAMT1-catalyzed the shift of free IAA to MeIA.

The regulatory mechanism of the homeostatic level between IAA and MeIAA has not been well documented, in which the issue how *IAMT1* is modulated is still now unclear. Our data provides a feasible way to understand this issue. *IAMT1* is induced by transgenic *TaWRKY71-1* in *Arabidopsis* (Fig. 7), while TaWRKY71-1, alike its homologue in rice, has no transcriptional activation activity in yeast (Fig. 4). This suggests that there should have at least one gene in the *TaWRKY71-1*–*IAMT1* regulatory cascade, and this gene could be inhibited by *TaWRKY71-1* and serve as a suppressor of *IAMT1*. The transcription level of *IAMT1* is strongly positively correlated to that of transgenic *TaWRKY71-1*, indicating that the regulatory effect of *TaWRKY71-1* on *IAMT1* is substantially accomplished at transcriptional-translational (direct linear correlation between the abundance of transcripts and proteins) but not post-translational (not due to protein modification-mediated activity modulation) level. The component(s) in the putative *TaWRKY71-1*–*IAMT1* regulatory cascade are, at least very likely, transcription factor(s), which transduce the *TaWRKY71-1′*s order in a single-threaded manner. The combination with screening *TaWRKY71* target genes and the transcription factors directly regulating *IAMT1* will help understand the regulatory mechanism of *IAMT1*-mediated leaf hyponasty.

Leaf curvature has proved to owe to the asymmetric cell elongation (cell size) and proliferation (cell number) between adaxial and abaxial sides of leaves. However, how *IAMT1*-mediated alteration of auxin homeostasis from free IAA to MeIAA causes hyponastic leaves is not discussed. *TaWRKY71-1* does not affect cell sizes and densities of both upper, lower epidermises and palisade mesophyll cells, but tightens spongy mesophyll tissue (Fig. 6). It was found that overexpression of E2F transcription factor in tobacco causes epinastic leaves, which is due to an accelerated proliferation of adaxial palisade mesophyll cells in the outer region and by repressed cell division on the abaxial spongy mesophyll side [29]. The mutant of auxin-associated gene *STENOFOLIA*, encoding a WUSCHEL-like homeobox transcriptional regulator, in *Medicago truncatula* leads to the diminishment of the difference in shape between palisade mesophyll cells (adaxial side) and spongy mesophyll cells (abaxial side) [30]. Together with these findings, our results suggest that auxin homeostatic level could substantially modulate leaf curvature development by changing the structure of spongy mesophyll tissue, showing the complicated anatomical basis of leaf hyponasty.

Unlike IAA–sugar and IAA–amino acid conjugates, MeIAA is essentially nonpolar, so it has a more efficient uptake than free IAA through diffusing through membranes rather than requiring an active transport system. This offers IAA esters the auxin activities similar to free IAA and in some cases even more potent than free IAA. Auxin response factors are crucial components in auxin signaling pathway and are often induced by increased auxin level [31]. However, *ARF8*, which has been found to be induced by auxin and influences cell expansion and proliferation during petal growth [32], is restricted by *TaWRKY71-1* in a transcription level dependent behavior (Fig. 7). One putative explanation is that apart from their similarities, MeIAA and free IAA may perform regulatory functions in different ways as well. Moreover, *TaWRKY71-1* induced the expression of *TCP24* and *HASTY* (Fig. 7), two *IAMT1*-restricted leaf curvature genes [18]. This implies that *TaWRKY71-1* may control the expression of these genes independent of IAA-MeIAA homeostasis. Besides, a novel transcriptional repressor TIE1 was recently proposed to serve as an inhibitory modulator of TCP activities, and its overexpression leads to hyponastic leaves [33]. We found that the promoter of *TIE1* possesses W-box that is specifically bound by WRKY transcription factors, suggesting *TaWRKY71-1* possibly also modulate TCPs via regulating *TIE1*. These speculations sound interesting and worthy of being elucidated.

In summary, we isolated a wheat WRKY transcription factor gene *TaWRKY71-1* that cause hyponastic leaves in *Arabidopsis* through modulating IAA and MeIAA levels. Our results provide a molecular clue to know how *IAMT1*-mediated auxin homeostasis is regulated. Moderate hyponasty of leaves is an important aspect of ideal plant type for crop yield improvement. Thus, *TaWRKY71-1* could be used for improving plant type in crop molecular breeding.
